# How Supplementation with SOD-Rich Plant Extract, Combined with Gliadin, Can Affect Oxidative Stress Markers and Zonulin Levels in Exercise-Induced Oxidative Stress

**DOI:** 10.3390/metabo13121200

**Published:** 2023-12-17

**Authors:** Olina Dudašova Petrovičova, Ivan Stanković, Brižita Ðordević, Violeta Dopsaj, Neda Milinković, Milivoj Dopsaj

**Affiliations:** 1 Faculty of Pharmacy, University of Belgrade, Vojvode Stepe 450, 11221 Belgrade, Serbia; istank@pharmacy.bg.ac.rs (I.S.); brizitadjordjevic@gmail.com (B.Ð.); nedan@pharmacy.bg.ac.rs (N.M.); 2 Faculty of Sport and Physical Education, University of Belgrade, Blagoja Parovića 156, 11000 Belgrade, Serbia; milivoj.dopsaj@gmail.com

**Keywords:** oxidative stress, athletes, superoxide dismutase, supplementation, zonulin

## Abstract

A randomized, double-blind, placebo-controlled study was conducted to *investigate* the influence of supplementation with a superoxide dismutase (SOD)-rich plant extract on markers of oxidative stress, zonulin levels and the performance of elite athletes. Participants were 30 international-level rowers, divided into an experimental group (*n* = 15) and a control group (*n* = 15). The rowers performed a maximal effort incremental test on a rowing ergometer at the beginning and at the end of the study. Markers of oxidative stress (total antioxidant status (TAS), total oxidant status (TOS), oxidative stress index (OSI), superoxide dismutase (SOD), glutathione peroxidase (GPx), advanced oxidation protein products (AOPPs), malondialdehyde (MDA), sulfhydryl (SH) groups, bilirubin, uric acid, albumin and zonulin) were determined in serum. A lower TOS (*p* = 0.010) and OSI (*p* = 0.004), a lower MDA (*p* = 0.001) and a higher level of SH groups (*p* = 0.031) were observed in the experimental group after supplementation. Physical performance was evaluated through metabolic efficiency, taking lactate levels and power output on the ergometer into account. After 6 weeks of supplementation, the relative increase in metabolic efficiency at a 4 mmol/L lactate concentration and maximal effort was significantly higher in the experimental group (*p* = 0.004 and *p* = 0.015, respectively). These results suggest that supplementation with a SOD-rich extract promotes lower oxidative stress, better antioxidant protection and, consequently, the better work performance of athletes.

## 1. Introduction

Oxidative stress is a phenomenon that has been the focus of numerous studies in various fields of science in recent decades. Oxidative stress represents an imbalance between oxidants and antioxidants in favor of oxidants. Oxidants have the properties of free radicals that have one or more unpaired electrons in their atomic orbital, which makes these compounds very reactive. The most common oxidants in aerobic organisms are reactive oxygen species (ROS). Various oxidants are synthetized during normal essential metabolic processes in the human body, but there are also many external causes that increase the chance of oxidative stress occurrence, including exposure to radiation, industrial chemicals, air pollutants, cigarette smoking, ozone, certain drugs and pesticides [1]. Besides this, strenuous exercise can also trigger a higher level of ROS synthesis. Elevated oxygen consumption during exercise contributes to a rise in ROS production by various cells and tissues, dependent on the exercise intensity, duration and training status of athletes [2]. High levels of ROS are associated with increased lipid peroxidation, glutathione oxidation, cellular lipids, proteins and DNA damage [3]. Oxidative stress is therefore connected to many undesirable processes in the human body, including aging processes; acquired immune deficiency syndrome; atherosclerosis; inflammatory diseases; the development and deterioration of some chronic diseases, such as cardiovascular and neurodegenerative diseases; cataracts; diabetes; and even cancer [1]. Exercise-induced oxidative stress is common in elite athletes due to intense training sessions and insufficient rest time, especially during the preparation and competition periods. Overtraining syndrome, a condition characterized by a higher injury frequency and lower athletic performance, is associated with elevated levels of oxidative stress biomarkers [4]. However, repetitive exercise can lead to the increased expression of antioxidant enzymes, regulated by higher levels of free radicals, leading to a stronger antioxidant defense system in athletes [5,6]. However, this adaptation mechanism often fails during periods of intense training [7,8]. Therefore, strategies of carefully planned training and an adequate diet for athletes frequently take into account additional antioxidant supplementation. There are numerous research papers related to antioxidant supplementation in sport, mostly with non-enzymatic antioxidants, including vitamins, minerals and phytochemicals, but much less with enzymatic antioxidants. Mostly, the antioxidant activity of plants is related to the occurrence of polyphenols naturally synthetized in various plant species. Quercetin is an antioxidant classed as a flavonoid and is found in foods such as red onion, dill and apple. It has been demonstrated that quercetin can encourage mitochondrial growth and reduce the perceived effort of exercise. Resveratrol is a naturally occurring polyphenol found in red grape and is thought to be responsible for many of the health benefits of the Mediterranean diet. Resveratrol, as with some other polyphenols, can induce mitochondrial biogenesis, which has subsequently been shown to enhance endurance capacity. Beetroot juice contains various phytochemicals, including betalain and polyphenols, which are from the anthocyanin and flavonoid subclass. Compared with that of other vegetables, the polyphenol content of beetroot is high. Beetroot juice also contains nitrate. It has been proven than nitrate content can contribute some performance benefits [9]. Chokeberry juice supplementation in a group of rowers limited exercise-induced oxidative damage to red blood cells, most probably by enhancing their endogenous antioxidant defense system due to its high anthocyanin content [10]. Also, artichoke extract has antioxidant properties thanks to its high content of chlorogenic acid, cynarine and flavonoids—derivatives of luteolin, and it is used in athletes’ supplementation [11].

Cucumis melo L.C. (Cucurbitaceae) is a plant naturally rich in SOD, and it is used in the formulation of an enzymatic antioxidant supplement containing melon extract combined with a gastro-resistant delivery system made of a biodegradable polymer (wheat gliadin), named GliSODin. Superoxide dismutase (SOD) is an essential part of our enzymatic antioxidant defense system. Joe McCord and Irwin Fridovich were the first scientists to describe the enzymatic activity of SOD and suggest its essential role in the defense system against free radicals, especially reactive oxygen species (ROS) [12]. Superoxide dismutase is a metalloenzyme present in three isoforms in humans: cytosolic copper–zinc SOD (SOD1), mitochondrial manganese SOD (SOD2) and extracellular copper–zinc SOD (SOD3) [13]. The main role of SOD is to catalyze the conversation of superoxide anion O_2_^•−^, a very reactive free radical molecule, to the less reactive hydrogen peroxide H_2_O_2_, further catabolized by catalase (CAT) and glutathione peroxidase (GPx) into H_2_O and O_2,_ thus preventing their further oxidation activity. Gliadin is utilized in this combination to provide gastro-resistance, but there are studies that have reported gliadin as being one of the most powerful triggers for zonulin release [14,15]. Zonulin is a physiological mediator known to regulate intestinal permeability reversibly by modulating intercellular tight junction openings [16]. It is used as a biochemical marker of increased gastric permeability. Nevertheless, strenuous exercise can trigger gastrointestinal problems, which may be a serious problem for athletes. A temporary disruption of splanchnic circulation can be caused by blood redistribution to peripheral tissue, which demands a higher level of oxygen supply during exercise. After intense exercise, when normal circulation is re-established, there is a large influx of oxygen to hypoxic gastrointestinal tissue, which can trigger higher ROS production, inflammation and mucosa damage, known as ischemia–reperfusion injury (IRI). These processes lead to enhanced gastrointestinal permeability and an enhanced zonulin level [17,18,19]. For these reasons, we investigate how 6 weeks of supplementation with a SOD-rich plant extract combined with gliadin can influence zonulin levels in athletes.

To the best of our current knowledge, there are only a few studies concerning the benefits of supplementation with GliSODin in athletes. The results of these studies suggest that supplementation can enhance antioxidant status [20] and reduce the level of oxidative stress [21], inflammation [20,22] and muscle damage [21,22]. Based on these findings, the main hypothesis was that supplementation with GliSODin will reduce the level of exercise-related oxidative stress. So, the goal of the current study was to examine in more detail the influence of a SOD-rich melo extract combined with gliadin supplementation on oxidative stress markers and the antioxidant response after forced training and on the parameters of sport performance and zonulin levels in elite athletes.

## 2. Materials and Methods

A randomized, double-blind, placebo-controlled study was conducted in accordance with the guidelines of the Declaration of Helsinki. The study was approved by the Ethics Committee of the Faculty of Pharmacy, Belgrade University (Protocol No. 2192/2). It is also on the Australian New Zealand Clinical Trials Registry (www.anzctr.org.au, accessed on 21 February 2022), study registry number: ACTRN12622000319774. The study protocol and the nature of the study were explained verbally and in written form to all participants. Written informed consent was obtained from the participants before the start of the study.

### 2.1. Subjects

Thirty (30) international-level rowers were recruited as subjects for this study. The sample size was determined using G-power software, version 3.1.9.4. All participants met the following inclusion criteria: healthy athletes, 18 to 30 years old and either gender. The exclusion criteria were as follows: gluten intolerance, allergy to any of the ingredients of the tested supplement and intake of other antioxidant supplements two weeks prior to the study. All participants were in good health and had no chronic medical conditions, such as diabetes or cardiovascular, gastrointestinal or kidney disease, or a surgical procedure in the previous 6 months that could interfere with the training regime. The athletes were asked not to take any other dietary supplements during the study and to inform the scientific staff if they had taken any other dietary supplements or medication during the study. The rowers were advised to stick to their regular eating habits.

### 2.2. Experimental Procedure

The athletes were randomly assigned to one of two groups. The experimental group (*n* = 15) received 2 GliSOdin capsules (2 × 250 mg) daily for 6 weeks, while the control group (*n* = 15) received 2 placebo capsules. The applied dose of the dietary supplement was determined according to the recommendations of the manufacturer. The placebo and GliSODin capsules were identical in appearance and taste. The placebo capsules contained maltodextrin instead of the active ingredient and magnesium stearate, mannitol and silicon dioxide as excipients. The GliSODin capsules contained 250 mg of concentrated melon extract naturally rich in SOD (Cucumis Melo L.C., Cucurbitacea), with an enzyme activity of 1mg GliSODin = 1IU SOD, combined with biodegradable protein gliadin, microcrystalline celluloses and magnesium stearate as excipients, manufactured by ISOCELL NUTRA S.A.S (Paris, France). A list of the ingredients of GliSODin can be found in Table 1. The combination of the enzyme and gliadin isolated from wheat is gastro-resistant, so it should ensure efficient bioavailability of the orally ingested enzyme [23]. The rowers took their supplement or placebo capsules one hour prior to training or one hour prior to breakfast on non-training days. The manufacturer recommended taking the supplement on an empty stomach. So, we agreed that one hour before training is a good time to take the supplement, as it may ensure the neutralization of the reactive species formed during training. The athletes were asked not to eat before training, which was in line with their usual meal plan recommendations. Supplementation was conducted during the mesocycle of baseline preparation with the usual training schedule and lasted 6 weeks.

The study protocol was designed to test the effect of supplementation on the oxidative stress induced by strenuous exercise, as well as on the baseline level of oxidative stress parameters during the 6-week supplementation. To assess the effect of GliSODin supplementation after intense exercise, a rowing ergometer was used for tests, in which all participants were tested to exhaustion on the first day at the beginning of the study and at the end of the supplementation period (after 6 weeks). Before the ergometer test, the rowers had a 24-h rest period as part of recuperation. On the rowing ergometer, an increased interval step test protocol was used according to a previously published study [22]. The test protocol was as follows: Rowers rowed 6 × 2 min with 5 min rest between the first two sets, 6 min between the second two sets and 8 min rest prior to the final session attempts. The rowing intensity was 150 W (watts), 200 W, 250 W, 300 W, 400 W and individual maximal effort for males, and 150 W, 180 W, 220 W, 250 W, 300 W and individual maximal effort for female rowers. 

The rowers followed a training schedule set by their coaches during the 6-week supplementation period. They trained 6 days per week, with each training session lasting an average of 2.05 h. 

**Figure 1:**
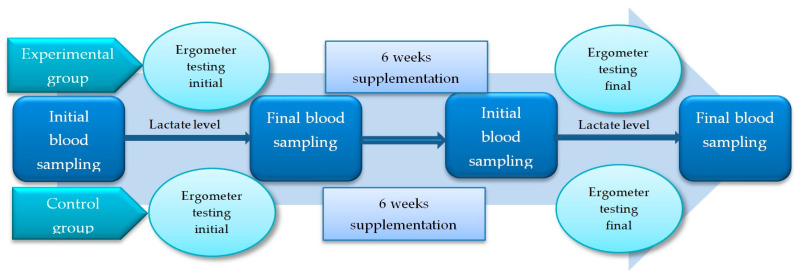
Study protocol.

Venous blood samples were taken on two occasions, both before the ergometer tests at the beginning of the study (initial test) and at the end of the study (final test). The first blood samples (initial blood sampling) were taken in the morning hours at rest, 20 min before the warm-up on the rowing ergometer. Secondly, blood samples were taken 10 min after testing on the ergometer until exhaustion in order to observe the changes in the blood parameters related to intensive exercise. During the sport performance testing, the rowers were only allowed to drink water. The lactate level in the capillary blood was also monitored during the tests. The baseline values were determined before training, and the rise in lactate levels was tracked 1 min after each 2-min rowing session. The same blood sampling was performed at the final testing. A schematic of the study protocol is shown in Figure 1.

Blood samples were taken from the antecubital vein and collected in BD Vacutainer^®^ tubes without additives for serum separation. Serum was separated via centrifugation at 3000 rpm for 10 min, and aliquots were stored at −20 °C or −80 °C until analysis.

### 2.3. Measurement 

In this study, we analyzed several prooxidant and antioxidant parameters as specific markers of free radical damaging activity. As markers of the oxidative/antioxidative status, we measured the total oxidative status (TOS), total antioxidative status (TAS) and oxidative status index (OSI). 

The total oxidative status (TOS) was determined using a spectrophotometric method by Erel [24]. Ferrous ion-o-dianisidine complexes are oxidized into ferric ions by the oxidizing agents present in the serum, and the reaction is enhanced by glycerol. The ferric ions form a colored complex with xylenol orange in an acidic medium. The color intensity is measured spectrophotometrically at 540 nm and correlates with the total number of oxidant molecules present in the sample. The assay is calibrated with hydrogen peroxide, and the results are expressed as micromolar hydrogen peroxide equivalent per liter (μmol H_2_O_2_ equiv./L); in our study, it was calculated as mmol H_2_O_2_ equiv./L, and TAS was calculated in the same way to be able to determine their ratio.

The total antioxidant status (TAS) in serum was determined using an automated measurement method based on the decolorization of the characteristic color of a 2,2′-azino-bis[3-ethylbenzthiazoline-6-sulfonic acid] (ABTS) radical cation by the antioxidants present in the sample. The intensity of the color change was measured using an Olympus AU-400 Biochemical Analyzer (Beckman Coulter INC. Brea, CA, USA). Trolox (a water-soluble analog of vitamin E, 6-hydroxy-2.5.7.8-tetramethylchroman-2-carboxylic acid) was used to calibrate the reaction rate. The TAS values of the samples are expressed as mmol Trolox equivalent/L [25].

The oxidative stress index (OSI), as a predictor of the antioxidant/prooxidant balance, was calculated as the ratio between TOS and TAS.

The MDA (malondialdehyde) level was determined using a thiobarbituric acid-reactive substance (TBARS) spectrophotometric assay based on the maximum absorbance of MDA and other TBARSs at 535 nm, as previously described by Girotti et al. [26].

AOPPs (advanced oxidation protein products) were determined spectrophotometrically at 340 nm under acidic conditions in the presence of potassium iodide according to the method previously described by Witko-Sarsat et al. [27]. AOPP concentrations were calibrated with chloramine-T solution and are expressed as μmol/L of chloramine-T equivalents. 

The concentrations of sulfhydryl groups (SH groups) in plasma were determined spectrophotometrically according to the method developed by Ellmann [28], in which the reaction of SH groups with 0.2 mmol/L 5,5′-dithiobis (2-nitrobenzoic acid) (DTNB) is followed by a color intensity change at 412 nm. 

Uric acid (UA) and total bilirubin in serum were measured in µmol/L using the spectrophotometric method with commercial reagents on the Olympus AU-400 Biochemical Analyzer (Beckman Coulter INC., Brea, CA, USA). Albumin was determined using the same biochemical analyzer and is expressed as g/L.

The serum Cu/Zn superoxide dismutase concentration was measured using a commercially available ELISA kit (Abcam, Boston, MA, USA) according to the manufacturer’s instructions. A spectrophotometric microplate reader (T-6100, Life and Analytical Sciences, Rayto, Shenzhen, China) was used to determine the SOD concentration at 450 nm. We used a high-sensitivity kit (the detection limit was 0.04 ng/mL), and the concentration of SOD is expressed as ng/mL. 

The concentration of glutathione peroxidase in serum was determined using a commercial ELISA kit (Abcam, Boston, MA, USA). The reaction mixture contained 40 mM NADPH, glutathione and glutathione reductase in assay buffer, and the reaction was started by adding cumene hydroperoxide. The concentration was calculated according to the degree of oxidation of NADPH to NADP+ under the assay kit conditions per minute. The change in absorbance was monitored spectrophotometrically at 340 nm, and the GPx concentration in serum is expressed as mU/mL.

The lactate (La) concentration in mmol/L was measured in capillary blood samples from the fingertip with a Lactate Plus portable test device using appropriate Nova Biomedical test strips. Samples were taken during both ergometer tests. The initial lactate level was determined before the test, and the increase in lactate concentration was monitored 1 min after each 2-min rowing session. 

The possible occurrence of hemolysis due to intensive physical activity was examined using the LIH test. This is a photometric test for a semi-quantitative assessment of lipemia/turbidity, icterus and hemolysis (LIH) in human serum and plasma using Beckman Coulter AU analyzers. Each sample tested showed a low level of interference below the critical limit concentration for each chromogen considered. 

The maximal power output expressed in W (watts) during the maximal effort attempt on the rowing ergometer, divided by the La concentration measured in the 1st minute after the ergometer test, represents the metabolic efficiency at the maximal tested power (Met.Eff.at max power). The rowing power at OBLA (the onset of blood lactate accumulation) as a characteristic threshold of lactic acidosis at 4 mmol/L and rowing power at 15 mmol/L as a characteristic threshold of a “sport-specific” post-race lactate concentration were calculated using a mathematical modeling procedure according to previously published methods [22,29,30,31]. The rowing performance at these characteristic cut-off values, 4 and 15 mmol/L lactate concentrations, was used to calculate metabolic efficiency variables as the ratio of watts achieved to lactate according to previously published research [22,29,30,31]. All participants were familiarized with the laboratory exercise test procedures prior to testing. A sport-specific performance test, i.e., a rowing test, was performed on a wind-resistance-braked rowing ergometer (Model D; Concept2, Morrisville, VT, USA). Ten minutes before each test session (initial and final tests), all rowers completed a five-minute warm-up program on the same ergometer. The rowing power achieved during the test was tracked for all participants on the ergometer monitor (expressed in watts).

### 2.4. Statistical Analyses

All statistical analyses were performed using IBM SPSS v 26.0 software. The anthropometric and training data of the study groups were compared using the ANOVA test. The influence of dietary supplementation and the time of measurement (before (T1) and after (T2) the ergometer test at baseline and before (T3) and after (T4) the final ergometer test) on the measured parameters was determined using linear mixed models with the Toeplitz covariance matrix statistical test, assuming the participants as a random effect and the experimental group and time as fixed effects. The relative values of the studied variables corresponding to the differences in changes with respect to the tests (within and between groups) are expressed as delta values in % (ΔTAS, ΔTOS, ΔOSI, ΔUA, Δbilirubin, Δalbumin, ΔSOD, ΔGPx, ΔAOPP, ΔSH, ΔMDA). For the calculation of all delta values, the following formula was used: ((T2/T1)-100) × 100, where T1 is the value of a particular variable before the test for a particular participant, and T2 is the value of the same variable for the same participant after the ergometer test. The differences for all variables determined via the calculation of delta values were calculated using the ANOVA repeated measure statistical test. The statistical significance level between the variables was set at *p* ≤ 0.05 [32].

## 3. Results

### 3.1. Anthropometric and Training Data of Study Participants

According to the data presented in Table 2, there were no significant differences between the tested groups in terms of average age, height, body weight and body mass index (BMI). The years of training and the average duration of daily training were also similar in both groups. 

### 3.2. Biochemical Parameters

The changes in the measured biochemical parameters of the prooxidative/antioxidative status during the initial test before supplementation and the final test after supplementation are shown in Table 3. There was a significant influence of time on the TAS value (*p* = 0.046), with a significantly lower TAS measured after the ergometer test at the end of the study after supplementation, without differences between the groups. The experimental group had lower values for TOS (*p* = 0.010) and OSI (*p* = 0.004) at the end of the study, and the time of measurement also had a significant impact on the OSI value (*p* = 0.026). 

**Figure 2:**
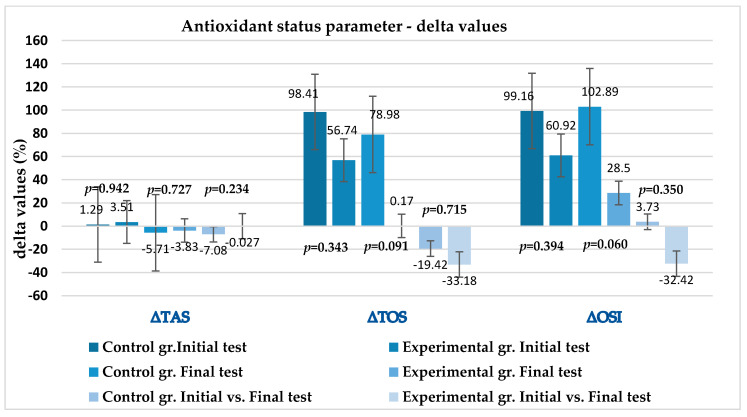
Changes in measured antioxidant status parameters due to specific maximal effort physical activity according to tests and experimental groups, expressed as delta values (in % of differences). Every *p*-value is related to the two adjacent bars, showing the change in value in groups concerning the tests; *p* < 0.05, statistical significance. ΔTAS—delta total antioxidant status; Δ TOS—delta total oxidant status; ΔOSI—delta oxidative stress index.

Figure 2 shows the results for the relative changes in the oxidative status parameters under the influence of intensive exercise and 6 weeks of supplementation within the groups. The results are expressed as delta values, which represent the percentage of change in the measured parameters in relation to the exercise (T1—before exercise; T2—after exercise) during the initial and final tests. In the initial tests, the values changed similarly in both groups, with no significant difference (ΔTAS% *p* = 0.942; ΔTOS% *p* = 0.343; and ΔOSI% *p* = 0.394). After 6 weeks of supplementation, the changes in the parameters caused by the ergometer test within the groups showed a marginally significantly higher ΔOSI% (*p* = 0.060) in the control group. In the figure, the last two bars for all three groups show the overall effect of supplementation on the measured parameters, presented as delta values (in %), between the tests (initial and final tests), taking into account the variable change within the groups (experimental and control). There was no significantly different effect on the oxidative status parameters in the initial and final tests (ΔTAS% *p* = 0.234; ΔTOS% *p* = 0.715; and ΔOSI% *p* = 0.350) within the two groups examined.

The changes in some non-enzymatic antioxidants in serum are shown in Table 4. A significant influence of the time of measurement on uric acid levels could be observed. There was a significant difference between the UA level at T1 vs. T2 and T1 vs. T4, but there was no influence of group. Total bilirubin was lower in the experimental group regardless of the time of measurement. The albumin level was similar in both groups, but the changes in the albumin concentration before and after the training reached significance (T1 vs. T2, T1 vs. T4, T2 vs. T3, T2 vs. T4 and T3 vs. T4).

Serum enzymatic antioxidant levels were assessed via changes in SOD and GPx concentrations due to exercise and supplementation (Table 5). The time of measurement in the tests had a significant effect on SOD levels (*p* = 0.001). The SOD concentration at the beginning of the study before supplementation (T1) was significantly lower than the SOD level after the test before supplementation (T2) and before (T3) and after the ergometer test after supplementation (T4). The SOD level after the final ergometer test (T4) was also significantly higher than that before the ergometer test (T3). There was no influence of group, but the influence of group and time together was significant (*p* ˂ 0.001). The time of sampling had a significant influence on the GPx level (*p* = 0.028); the GPx level before the ergometer test at the end of the study was lower than the level after the test at the beginning of the study (T2 vs. T3).

The results of the changes in the oxidative stress parameters are shown in Table 6. Dietary supplementation and training had no effect on the AOP values. However, the experimental group had a higher SH value regardless of the time of measurement (*p* = 0.031), but there was a marginal significant effect of group and time together on the SH value (*p* = 0.059). Both time (*p* ˂ 0.001) and group (*p* = 0.001), as well as time and group together (*p* ˂ 0.001), had a significant influence on the MDA values during the study. 

**Figure 3:**
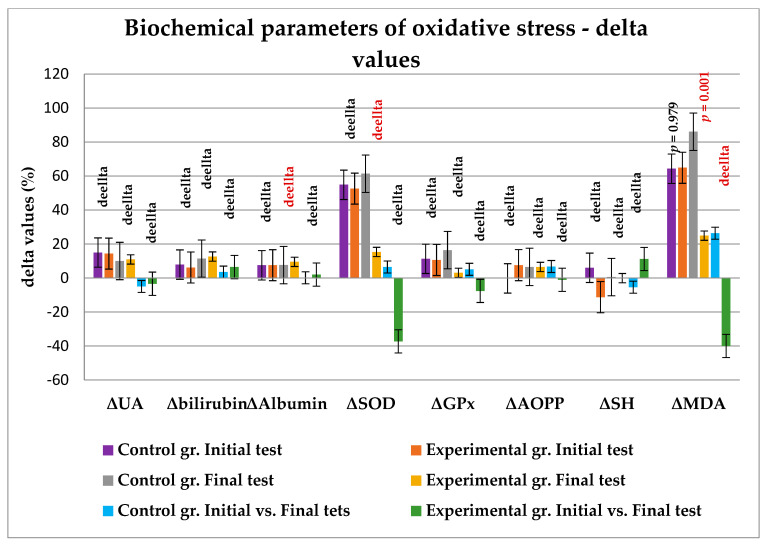
Changes in measured biochemical parameters of oxidative stress due to specific maximal effort physical activity according to tests and experimental groups, expressed as delta values (in % of differences). Every *p*-value is related to the two adjacent bars, showing the changes in values in groups concerning the tests; *p* < 0.05, statistical significance. ΔUA-delta uric acid; ΔSOD-delta superoxide dismutase; ΔGPx-delta glutathione peroxidase; Δ AOPP-delta advanced oxidation protein products; ΔSH-delta total sulfhydryl groups; Δ MDA-delta malondialdehyde.

The effects of intensive physical activity and supplementation on the relative changes in the biochemical parameters within the groups are shown in Figure 3. The results are expressed as delta values presenting the changes in the measured parameters in percentage relative to the exercise (T1—before exercise; T2—after exercise) during the initial and final tests. There were no statistically significant changes in the oxidative stress parameters (ΔUA *p* = 0.811; Δ bilirubin *p* = 0.670; Δalbumin *p* = 0.969; ΔSOD *p* = 0.908; Δ GPx *p* = 0.927; ΔAOPP *p* = 0.168; ΔSH *p* = 0.167; and ΔMDA *p* = 0.979) between the groups in the first test before supplementation (the first two bars for each group). The changes in some parameters in the groups at the end of the study were significantly affected by the strenuous exercise on the rowing ergometer, including the changes in Δalbumin (*p* = 0.054), ΔSOD (*p* = 0.011) and ΔMDA (*p* = 0.0001). The delta differences (in %) between the tests (initial and final tests) according to the variables and considering the groups (experimental and control) are shown as the fifth and the sixth bars in every bar grouping. The increase in the MDA value in the control group reached statistical significance (*p* = 0.018). The changes in the other values were not significant.

As for the zonulin levels, they increased in both the initial and final tests after the strenuous training on the rowing ergometer in both groups, without significant differences between the groups. A significant difference was calculated between the zonulin levels at T1 vs. T4, T2 vs. T4 and T3 vs. T4 (Table 7).

Table 8 shows the delta values of the changes in zonulin levels within the groups after the initial and final tests and overall during the study as percentages of the changes. The changes did not reach statistical significance.

Table 9 shows the effects of supplementation on the rowers’ work performance. There were no differences between the groups in terms of metabolic efficiency at all three observation points in both ergometer tests. 

**Figure 4:**
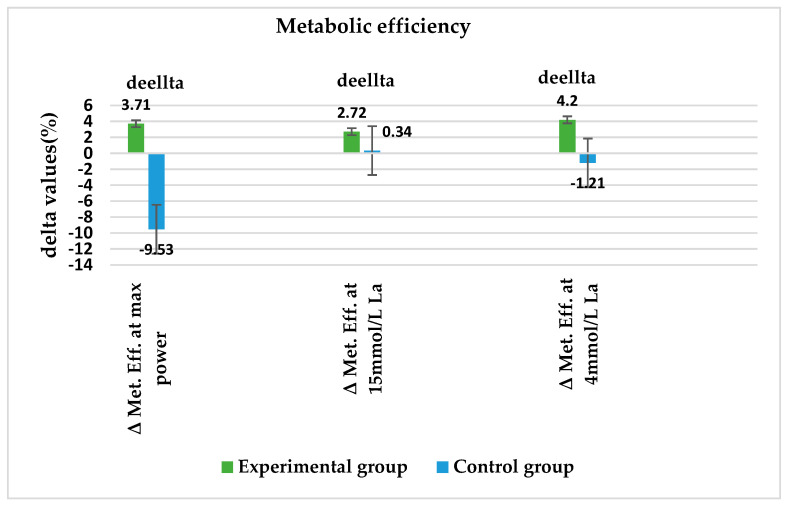
Descriptive results considering explored variables of work performance according to the tests and supplementation groups, expressed as delta values (in % of differences); *p* < 0.05, statistical significance.

By examining the percentage changes in the work performance variables examined within the groups, we observed that there were significant differences between the groups in terms of the change in metabolic efficiency during the supplementation period. The metabolic efficiency at the maximum tested power was 3.71% higher in the experimental group and 9.53% lower in the control group after supplementation, and this difference was significant (*p* = 0.015). The metabolic efficiency at 4 mmol/l La was also significantly higher in the supplemented group than in the control group (*p* = 0.004), where it was lower at the end of the study, as shown in Figure 4.

## 4. Discussion

The main objectives of the current study were to investigate the antioxidative effects of supplementation with a plant extract rich in SOD combined with gliadin, called GliSODin, and to investigate the possible positive effects on the work performance of elite athletes. It is well documented that intense physical activity can cause metabolic stress [2,3,33]. Elite athletes who train excessively and for long periods of time are more susceptible to exercise-induced oxidative stress and the potential damage caused by it. Therefore, under these conditions, dietary antioxidants may play an important role in maintaining a desirable prooxidant/antioxidant balance. The concentrations of different oxidant species in serum can be measured separately, but TOS represents the sum of peroxides, including hydrogen peroxide and various lipid peroxides, in serum, and it can be a good indicator of the oxidation status in the organism [25]. In our study, intense physical activity performed in an ergometer test until exhaustion led to an increase in the TOS and OSI levels in both groups of rowers, which is consistent with previous studies [34,35]. However, at the end of the study, after 6 weeks of supplementation, TOS had significantly decreased in the supplemented group (*p* = 0.010). The oxidative status index (OSI), which provides a better picture of the balance between prooxidants and antioxidants in serum, was also lower in the experimental group (p= 0.004). The relative increase in OSI in the control group in the final examination was close to statistical significance (*p* = 0.060). These results could be explained by the beneficial effects of GliSODin supplementation, which leads to lower levels of reactive oxidative species.

The total amount of non-enzymatic antioxidants in serum (measured via TAS) usually increases in response to acute exercise to maintain antioxidant status and protect the body from high levels of ROS [36,37], but the results are inconsistent and usually depend on the timing of blood sampling and the type of exercise. In our study, the specific maximal effort test on the rowing ergometer did not significantly alter the TAS levels in the groups of rowers tested, similar to some previous studies [38,39], but, after the exercise in the final test, the TAS levels were significantly lower in both groups.

To better assess the effects of supplementation, we measured the impact on the levels of some antioxidant parameters, such as albumin, uric acid and total bilirubin, as well as the antioxidant enzymes SOD and GPx separately.

Uric acid, as the end product of purine degradation, is one of the major components of the non-enzymatic antioxidant system and is thought to determine about 35–65% of TAS [40,41]. As expected, we found higher uric acid levels after exercise (*p* ˂ 0.001), but the difference between groups over the course of the study was insignificant. Bilirubin, the end product of heme degradation, exerts antioxidant effects due to the redox cycle in which it is oxidized to biliverdin by ROS and then recycled by biliverdinreductase. Strenuous exercise has been found to induce an increase in bilirubin [42], but, in our study, we found no differences in the tested rowers at the beginning or at the end of the study after exercise. The bilirubin levels were lower in the group of rowers during the study, and all values were within the reference range. Hypervolemia is a well-documented response to endurance training [43]. Albumin is responsible for ~75% of oncotic pressure in plasma due to its low molecular mass (69 kDa) and abundance in plasma. It is also an important ligand-binding and free radical scavenging circulating antioxidant [44]. The albumin level was higher in this study after each testing session (*p* ˂ 0.001), with no differences between the groups. However, the nearly significant increase in delta albumin levels in the experimental group (*p* = 0.054) in the final test may have contributed to better antioxidant protection. The lack of a greater increase in the measured circulating antioxidants could be explained by the timing of the measurement, 10 min after exercise. We would have obtained a better insight if the measurement had been performed over a longer period, such as 24 or 48 h after the test.

Considering that GliSODin is an enzymatic antioxidant supplement, containing SOD of plant origin, we hypothesized that supplementation will improve the enzymatic antioxidant system. The levels of the selected enzymes SOD and GPx increased after intensive training in both groups before supplementation (Table 5). However, after 6 weeks of supplementation, the SOD levels increased significantly in the supplemented group (*p* ˂ 0.001), which can be attributed to a combined effect of training and supplementation. These results can be considered evidence for the bioavailability of SOD from the combination of SOD-rich melon extract and gliadin, ingested orally. The GPx levels increased during the strenuous exercise with no differences between groups in the initial and final tests. These results are in line with the results of a study with Polish rowers who took the same dose of GliSODin [20]. In contrast, in a study with divers exposed to a 60-min hyperbaric treatment (2.5 ATA) supplemented with GliSODin (1000 IU), no effect on SOD levels was observed, and GPx levels were even lower in the supplemented group [45]. Observing the changes in the delta values of the enzyme concentrations within the groups (Figure 3), the relative increase in the SOD concentration (*p* = 0.011) was greater in the control group in the final test. This could be explained by an increased need for antioxidant protection in the control group due to the lower initial level of the enzyme. In this study, we did not follow the changes in SOD levels after the period of supplementation. We think that it would be an interesting avenue for further studies to measure SOD and GPx levels over a longer period of time after supplementation, for example, several weeks or even months.

The consequences of oxidative stress were assessed via the changes in AOPPs, total SH groups and MDA levels. The content of total thiol groups is an indirect indicator of serum glutathione levels. In a study with a group of female volleyball players, a higher level of SH groups was found depending on the years of training [46]. Other studies, in which the level of SH groups was observed within a short training period, could not confirm this increase [47]. In our study, the level of SH groups was significantly higher in the experimental group over the course of the study (*p* = 0.031), but the timing of the measurement in relation to the training in the initial test, as well as in the final test, did not influence SH levels significantly. When considering the influence of time and group together on the SH groups, we found marginal significance (*p* = 0.059). Although the specific maximal effort ergometer test and the supplementation used in this study had no effect on the level of protein oxidation products (AOPPs), a significant effect was found on the level of lipid oxidation measured via MDA. Elevated MDA levels are considered an exercise-induced oxidative stress marker [48]. There was a significant increase in MDA levels after each ergometer test in both groups, showing the influence of the time of measurement (*p* ˂ 0.001). There was also a significant difference between the groups (*p* = 0.001) and a significant effect of time and experimental group (*p* ˂ 0.001) on MDA levels, which were lower in the experimental group after the supplementation period. We found this to be a valuable result of the study. Skarpanska-Stejnborn et al. [20] demonstrated a significant increase in TBARS levels after a 2000-m rowing ergometer test, but GliSODin supplementation had no effect on TBARS levels. However, Arent et al. [21] found a significant decrease in LPO (lipid hydroperoxide), and Muth et al. [45] found a lower level of 8-isoprostane after GliSODin supplementation. The changes in these parameters indicate lower lipid oxidation, which could be attributed to GliSODin supplementation. In future studies, it would be interesting to compare the effect of supplementation on MDA levels as a marker of oxidative stress in cellular lipids with the changes in glutathione levels, an important antioxidant, during and after exercise.

Zonulin is a physiological modulator of the opening of intercellular tight junctions, which are involved in the transport of macro- and micro-molecules. It can therefore increase intestinal permeability [14]. This is the main reason why gliadin may help plant SOD to cross the intestinal barrier. We found no statistically significant differences between the control and experimental groups in terms of zonulin concentrations. These results should rule out a possible negative perspective of the interaction between gliadin and enterocytes leading to an undesirable increase in intestinal permeability considering GliSODin supplementation. We also examined how the zonulin level correlated with the values of the measured biochemical parameters at the end of the study after the ergometer test. This yielded some interesting findings. According to the Spearman correlation test, there was a significant positive correlation between CPR and zonulin levels (rho = 0.571, *p* = 0.026). However, there was a significant negative correlation between the zonulin level after the ergometer test and the GPx level before (rho = −0.564, *p* = 0.028) and after (rho = −0.764, *p* = 0.001) training. These results suggest that inflammation increases zonulin levels, while higher antioxidant protection may lead to lower zonulin levels, which is consistent with previous research results [11,14]. Other correlations did not reach significance.

A limitation of this study is that we measured the zonulin concentration in serum; for a better assessment, it should also be determined in stool samples. To confirm the effects of antioxidant supplementation on zonulin levels and intestinal permeability, a study with a larger number of participants, including athletes from different sports, and a longer duration of supplementation is needed.

To investigate the potential impact of supplementation on the rowers’ work performance in this study, we tracked metabolic efficiency at several points, such as the ratio of power output to lactate concentration. As described, we observed changes in metabolic efficiency at the maximal power output during the ergometer test at 4 mmol/L lactate concentration; this concentration is considered the onset of blood lactate accumulation (OBLA), and a 15 mmol/L lactate concentration is considered the mean value of the maximal lactate concentration at the end of a 2000 m race, which is typical for elite rowers. The delta value of metabolic efficiency at the maximal tested power showed an increase in the experimental group (3.71%) in contrast to a decreased value in the control group (−9.53%), and this difference was significant (*p* = 0.015). The relative change in metabolic efficiency at a lactate concentration of 4 mmol/L was significantly higher (*p* = 0.004) in the experimental group (4.2%) than in the control group (−1.21%). It seems that supplementation with GliSODin resulted in better metabolic efficiency in the experimental group in the specific ergometer test used in this study, indicating better work performance of the rowers.

## 5. Conclusions

Supplementation with GliSODin in a group of international-level rowers for a 6-week period resulted in a decrease in TOS, OSI and MDA and an increase in SOD levels and certain parameters of work performance. These results suggest that GliSODin is good nutritional support for athletes, leading to lower oxidative stress, better antioxidant protection and, consequently, better sport performance. A limitation of this study is the small number of participants and the short period of supplementation. In addition, it would be very interesting to investigate the effects of supplementation during the strenuous training period before and during the competition period under more controlled conditions. Our study was conducted during the basic preparation period between competitions. Although the currently available data and recent results are encouraging, more extensive, well-controlled clinical studies are suggested to confirm these beneficial effects of GliSODin in the elite athlete population.

## Figures and Tables

**Table 1:** GliSOD in ingredients.

Usual Name	Latin Binomial	Plant Part	CAS
Melon concentrate	*Cucumis melo* L.	Fruit pulp	90063-94-8
Gliadin	*Triticum vulgare*	Wheat grain	9007-90-3
Maltodextrin	*Triticum* spp.	Wheat grain	9050-36-6

**Table 2:** Anthropometric and training data.

Parameter	Experimental Group (*n* = 15)	Control Group (*n* = 15)	Differences
Age (years)	25.5 ± 5.43	22.1 ± 5.7	*p* = 0.098
Height (m)	1.86 ± 0.08	1.88 ± 0.08	*p* = 0.387
Body weight (kg)	86.4 ± 8.7	84 ± 11.2	*p* = 0.507
BMI (kg/m^2^)	23.6 ± 2.45	24.3 ± 1.71	*p* = 0.359
Years of training	8.5 ± 5.0	7.5 ± 5.6	*p* = 0.613
Training duration per day (hours)	2.05 ± 0.36	2.05 ± 0.46	*p* = 0.993

**Table 3:** Changes in oxidative status parameters during initial test before supplementation and final test after supplementation.

	Initial Test		Final Test					
Parameter	T1 (Before Test)	T2 (After Test)	T3 (Before Test)	T4 (After Test)	Total	Time	Group	Time × Group
**TAS (mmol Trolox equivalent/L)**								
Experim gr. (*n* = 15)	0.426 ± 0.042	0.408 ± 0.055	0.434 ± 0.056 *	0.412 ± 0.054 ^γ^	0.420 ± 0.053	*p* = 0.046	*p* = 0.146	*p* = 0.318
Control gr. (*n* = 15)	0.413 ± 0.048	0.417 ± 0.064	0.419 ± 0.046 *	0.384 ± 0.052 ^γ^	0.4082 ± 0.054			
**TOS (mmol H_2_O_2_ equiv./L)**								
Experim gr. (*n* = 15)	16.43 ± 9.71	20.71 ± 12.80	14.29 ± 6.23	17.14 ± 9.58	17.14 ± 9.88 ^†^	*p* = 0.091	*p* = 0.010	*p* = 0.393
Control gr. (*n* = 15)	15.87 ± 12.38	28.87 ± 29.78	22.85 ± 25.18	29.43 ± 22.11	24.25 ± 23.32			
**OSI (arbitrary units)**								
Experim gr. (*n* = 15)	3.87 ± 2.26	5.21 ± 3.13	3.29 ± 1.32	4.15 ± 2.21	4.13 ± 2.36 ^†^	*p* = 0.026	*p* = 0.004	*p* = 0.214
Control gr. (*n* = 15)	3.73 ± 2.53	6.90 ± 6.29	5.49 ± 5.96	7.77 ± 5.61	5.97 ± 5.40			

^γ^ *p* ˂ 0.050 relative to time 3 (T3), * *p* ˂ 0.050 relative to time 4 (T4), ^†^ *p* ˂ 0.050 relative to control group. TAS—total antioxidant status; TOS—total oxidant status; OSI—oxidative stress index.

**Table 4:** Changes in biochemical parameters of non-enzymatic antioxidants during initial test before supplementation and final test after supplementation.

	Initial Test		Final Test					
Parameter	T1 (Before Test)	T2 (After Test)	T3 (Before Test)	T4 (After Test)	Total	Time	Group	Time × Group
**UA (µmol/L)**								
Experim gr. (*n* = 15)	385.1 ± 79.6 ^α,^*	439.5 ± 90.0 ^#^	397.0 ± 105.8	4369 ± 106.3 ^#^	414.64 ± 96.65	*p* ˂ 0.001	*p* = 0.715	*p* = 0.127
Control gr. (*n* = 15)	361.5 ± 94.1 ^α,^*	414.4 ± 106.1 ^#^	433.8 ± 123.8	475.1 ± 1306 ^#^	421.21 ± 118.98			
**Total Bilirubin (µmol/L)**								
Experiml gr. (*n* = 15)	15 ± 7.6	16 ± 8.6	15 ± 7.4	17 ± 8.0	16.0 ± 7.8 ^†^	*p* = 0.141	*p*˂0.001	*p* = 0.920
Control gr. (*n* = 15)	17 ± 10.7	18 ± 12.3	16 ± 11.1	18 ± 12.0	17.5 ± 11.4			
**Albumin (g/L)**								
Experim gr. (*n* = 15)	45.6 ± 2.4 ^α,^*	49.0 ± 2.9 ^#,γ^,*	45.9 ± 2.4 ^α,^*	50.3 ± 2.6 ^#,α,γ^	47.7 ± 3.22	*p* ˂ 0.001	*p* = 0.814	*p* = 0.789
Control gr. (*n* = 15)	45.5 ± 2.2 ^α,^*	48.9 ± 2.1 ^#,γ,^*	46.5 ± 1.9 ^α,^*	50.0 ± 2.1 ^#,α,γ^	47.8 ± 2.32			

^#^ *p* ˂ 0.050 relative to time 1 (T1), ^α^ *p* ˂ 0.050 relative to time 2 (T2), ^γ^ *p* ˂ 0.050 relative to time 3 (T3), * *p* ˂ 0.050 relative to time 4 (T4), ^†^ *p* ˂ 0.050 relative to control group. UA—uric acid.

**Table 5:** Changes in enzymatic antioxidants during initial test before supplementation and final test after supplementation.

	Initial Test		Final Test					
Parameter	T1 (Before Test)	T2 (After Test)	T3 (Before Test)	T4 (After Test)	Total	Time	Group	Time × Group
**SOD (ng/mL)**								
Experim gr. (*n* = 15)	63.5 ± 60.0 ^α,γ,^*	84.9 ± 74.8 ^#^	121.0 ± 75.0 ^#,^*	127.3 ± 67.4 ^#,γ^	99.16 ± 72.94	*p* ˂ 0.001	*p* = 0.303	*p* ˂ 0.001
Control gr. (*n* = 15)	73.8 ± 37.4 ^α,γ,^*	111.8 ± 51.44 ^#^	61.3 ± 34.6 ^#,^*	96.5 ± 53.8 ^#,γ^	85.86 ± 48.17			
**GPx (mU/mL)**								
Experim gr. (*n* = 15)	189.5 ± 49.4	202.5 ± 40.4 ^γ^	176.2 ± 65.7 ^α^	177.1 ± 67.6	186.31 ± 56.50	*p* = 0.028	*p* = 0.601	*p* = 0.412
Control gr. (*n* = 15)	168.2 ± 61.9	182.7 ± 60.4	161.5 ± 60.4	179.4 ± 54.2	172.97 ± 58.43			

^#^ *p* ˂ 0.050 relative to time 1(T1), ^α^ *p* ˂ 0.050 relative to time 2 (T2), ^γ^ *p* ˂ 0.050 relative to time 3 (T3), * *p* ˂ 0.050 relative to time 4 (T4). SOD—superoxide dismutase; GPx—glutathione peroxidase.

**Table 6:** Changes in biochemical parameters of oxidative stress during initial test before supplementation and final test after supplementation.

	Initial Test		Final Test					
Parameter	T1 (Before Test)	T2 (After Test)	T3 (Before Test)	T4 (After Test)	Total	Time	Group	Time × Group
**AOPP (µmol/L)**								
Experim gr. (*n* = 15)	62.71 ± 16.54	67.34 ± 19.31	61.59 ± 14.91	65.54 ± 17.44	64.30 ± 16.84	*p* = 0.999	*p* = 0.226	*p* = 0.999
Control gr. (*n* = 15)	65.33 ± 20.83	66.29 ± 28.22	66.52 ± 23.25	72.01 ± 31.17	67.54 ± 25.65			
**SH (mmol/L)**								
Experim gr. (*n* = 15)	0.46 ± 0.14	0.39 ± 0.09	0.49 ± 0.10	0.48 ± 0.14	0.46 ± 0.12 ^†^	*p* = 0.120	*p* = 0.031	*p* = 0.059
Control gr. (*n* = 15)	0.41 ± 0.07	0.44 ± 0.18	0.43 ± 0.14	0.43 ± 0.16	0.43 ± 0.14			
**MDA (µmol/L)**								
Experim gr. (*n* = 15)	4.59 ± 2.16^α,*^	7.01 ± 3.26 ^#,γ^	3.72 ± 0.60 ^α,^*	4.55 ± 0.69 ^#,γ^	4.97 ± 2.32 ^†^	*p* ˂ 0.001	*p* = 0.001	*p*˂0.001
Control gr. (*n* = 15)	4.08 ± 1.02^α,*^	6.68 ± 2.29 ^#,γ^	4.45 ± 1.24 ^α,^*	8.03± 2.53 ^#,γ^	5.81 ± 2.46			

^#^ *p* ˂ 0.050 relative to time 1 (T1), ^α^ *p* ˂ 0.050 relative to time 2 (T2), ^γ^ *p* ˂ 0.050 relative to time 3 (T3), * *p* ˂ 0.050 relative to time 4 (T4), ^†^ *p* ˂ 0.050 relative to control group. AOPP—advanced oxidation protein products; SH—total sulfhydryl groups; MDA—malondialdehyde.

**Table 7:** Changes in zonulin concentration during ergometer testing.

	Initial Test		Final Test					
	T1 (Before Test)	T2 (After Test)	T3 (Before Test)	T4 (After Test)	Total	Time	Group	Time × Group
Zonulin (ng/mL)								
Experimental gr. (*n* = 15)	7.68 ± 7.43 *	9.36 ± 4.55 *	9.22 ± 2.86 *	13.46 ± 6.08 ^#,α,γ^	9.93 ± 6.57	*p* ˂ 0.001	*p* = 0.239	*p* = 0.804
Control gr. (*n* = 15)	7.49 ± 4.94 *	10.53 ± 5.61 *	11.44 ± 4.98 *	14.28 ± 7.89 ^#,α,γ^	10.94 ± 5.37			

^#^ *p* ˂ 0.050 relative to time 1 (T1), ^α^ *p* ˂ 0.050 relative to time 2 (T2), ^γ^ *p* ˂ 0.050 relative to time 3 (T3), * *p* ˂ 0.050 relative to time 4 (T4).

**Table 8:** Changes in zonulin concentration due to specific maximal effort physical activity according to tests and experimental groups, expressed as delta values (in % of differences).

	Experimental gr.	Control gr	*p* Values
Δ Zonulin % initial test	58.19 ± 65.26	73.15 ± 134.58	0.702
Δ Zonulin % final test	44.24 ± 41.27	30.30 ± 57.86	0.454
Δ Zonulin % initial vs. final test	−13.95 ± 65.25	−42.85 ± 147.19	0.493

**Table 9:** Changes in sport performance parameters during ergometer test before and after supplementation.

Parameter	Initial Test	Final Test	Total	Time	Group	Time × Group
**Met. Eff. at max power**						
Experimental gr. (*n* = 15)	29.55 ± 5.49	30.26± 5.21	29.91 ± 5.27	*p* =0.410	*p* = 0.565	*p* = 0.190
Control gr. (*n* = 15)	30.62 ± 5.56	27.56 ± 5.68	29.09 ± 5.74			
**Met. Eff. at 15 mmol/L La**						
Experimental gr. (*n* = 15)	29.84± 5.03	30.55± 4.82	30.19 ± 4.86	*p* = 0.986	*p* = 0.697	*p* = 0.594
Control gr. (*n* = 15)	30.04 ± 5.58	29.28 ±5.69	29.66 ± 5.55			
**Met. Eff. At 4 mmol/L La**						
Experimental gr. (*n* = 15)	67.67 ± 11.85	70.33 ± 11.87	69.00 ± 11.73	*p* = 0.104	*p* = 0.079	*p* = 0.341
Control gr. (*n* = 15)	68.47 ± 12.14	67.54 ± 11.79	68.00 ± 11.77			

## Data Availability

The data presented in this study are available on request from the corresponding author. The data are not publicly available due to privacy.
